# Development of loop-mediated isothermal amplification (LAMP) assay using SYBR safe and gold-nanoparticle probe for detection of *Leishmania* in HIV patients

**DOI:** 10.1038/s41598-021-91540-5

**Published:** 2021-06-09

**Authors:** Toon Ruang-areerate, Charanyarut Sukphattanaudomchoke, Thanyapit Thita, Saovanee Leelayoova, Phunlerd Piyaraj, Mathirut Mungthin, Patcharapan Suwannin, Duangporn Polpanich, Tienrat Tangchaikeeree, Kulachart Jangpatarapongsa, Kiattawee Choowongkomon, Suradej Siripattanapipong

**Affiliations:** 1 Department of Parasitology, Phramongkutklao College of Medicine, Bangkok, 10400 Thailand; 2 Department of Microbiology, Faculty of Science, Mahidol University, Bangkok, 10400 Thailand; 3 Center for Research and Innovation, Faculty of Medical Technology, Mahidol University, Bangkok, 10700 Thailand; 4 National Nanotechnology Center (NANOTEC), National Science and Technology Development Agency (NSTDA), Pathum Thani, 12120 Thailand; 5 Department of Biochemistry, Faculty of Science, Kasetsart University, Bangkok, 10900 Thailand

**Keywords:** Microbiology, Infectious-disease diagnostics, Parasitology

## Abstract

Asymptomatic leishmaniasis cases have continuously increased, especially among patients with HIV who are at risk to develop further symptoms of cutaneous and visceral leishmaniasis. Thus, early diagnosis using a simple, sensitive and reliable diagnostic assay is important because populations at risk mostly reside in rural communities where laboratory equipment is limited. In this study, the highly sensitive and selective determination of *Leishmania* infection in asymptomatic HIV patients was achieved using dual indicators (SYBR safe and gold-nanoparticle probe; AuNP-probe) in one-step LAMP method based on basic instruments. The assay can be simply evaluated under the naked eye due to clear interpretation of fluorescent emission of LAMP-SYBR safe dye-complex and colorimetric precipitate of specific AuNP-probes. The sensitivities and specificities of fluorescent SYBR safe dye and AuNP-probe indicators were equal, which were as high as 94.1 and 97.1%, respectively. Additionally, detection limits were 10^2^ parasites/mL (0.0147 ng/µL), ten times more sensitivity than other related studies. To empower leishmaniasis surveillance, this inexpensive one-step SYBR safe and AuNP-LAMP assay is reliably fast and simple for field diagnostics to point-of-care settings, which can be set up in all levels of health care facilities including resource limited areas, especially in low to middle income countries.

## Introduction

Leishmaniasis, a vector-borne disease caused by flagellate protozoa of the genus *Leishmania*, remains one of the global health challenges affecting millions of people worldwide. Disease manifestations range from self-healing cutaneous leishmaniasis (CL) to potentially fatal outcome, visceral leishmaniasis (VL)^1^. Since the emergence of CL and VL caused by *L. (Mundinia) martiniquensis* and *L. (Mundinia) orientalis* in northern and southern Thailand have been reported, the number of infected cases has continuously increased among patients with HIV/AIDS^2,3^. Notably, HIV probably influences the transmission of leishmaniasis in affected areas due to a decrease in host immunity.

Currently, polymerase chain reaction (PCR) is considered the primary diagnostic test for HIV coinfection cases because this method is trustworthy with high sensitivity and specificity^3,4^. However, time consuming PCR takes about 5 to 8 h and requires costly instruments to detect specific amplicons that becomes inappropriate for fieldwork^5^. On the contrary in terms of simplicity, the loop-mediated isothermal amplification (LAMP) assay, a simple approach based on isothermal conditions between 60 to 65 °C and a set of primers to amplify DNA by strand displacement activity of *Bst* polymerase, rapidly generates large amounts of DNA in a short time with high sensitivity and specificity^6–8^. Obviously, the technique is robust, reliable, cost effective and field-friendly overcoming the need of a thermal cycler^9^.

Several detection methods including turbidity, fluorescence and color have been developed to visualize and measure LAMP products^10,11^. However, ambiguous reading interpretations could sometimes occur between very pale blue color and transparency for colorimetric assays based on malachite green (MG) dye^12,13^. SYBR green I, a fluorescent indicator, which additionally requires postamplification preparation has been widely used due to clear interpretation under both ultraviolet (UV) and visible lights^14–18^. To avoid the common contamination prone in the LAMP assay during the postamplification preparation step, a fluorescent detection reagent (FDR) is alternatively used in a prereaction^1,16,19^. Nevertheless, inhibitory effects of SYBR green I on prereaction preparation could be overcome if the final dye concentration was lower than 0.4× or isolated dye during prereaction mixture preparation was performed, e.g., closed tube LAMP assays^15,18,20–23^. Recently, a low cost and nonmutagenic fluorescent dye, SYBR safe, was shown to reduce ambiguous evaluation without the need of postamplification preparation; however, the technique required a fluorescent light source to detect LAMP amplicons^13^.

The impact of gold nanoparticles (AuNPs) as a biomarker sensor for clinical diagnosis has been particularly relevant regarding selective binding of modulated ligand functionalization on the surface of AuNPs^24–26^. Simple and inexpensive methods based on bio-nanoprobes, Thiol-linking of DNA and chemical functionalization of AuNPs for specific protein/antibody binding, were commonly applied to detect specific DNA sequences and are presently being expanded to diverse fields of pathogen diagnosis^24,26–29^. Usually, oligonucleotide probes were labeled with AuNP offering the advantages of low cost and direct visual inspection^30–32^. After hybridization with complementary DNA targets, the red solution remains indicating positive results because the hybridized complex structure between the complementary targets and AuNP probes forms the polymeric network of cross-linked AuNPs, thus preventing them from aggregation by salt induction. On the other hand, aggregation of the AuNP probes due to noncrosslinking hybridization is induced by increasing salt concentration and results in a color change from red to blue (negative result)^33,34^. The size and distance of the dependent optical properties of these particles contribute to the color change in aggregations of AuNPs^35,36^. Thus, unambiguous interpretation of AuNP probes based colorimetric assay was simply favorable to be visually evaluated by the naked eye. This is preferable for the development of effortless, fast and feasible field detection methods, especially, for the molecular detection of infectious pathogens^29,37^.

In this study, we developed one-step LAMP assay based on dual indicators for detection of *Leishmania* DNA in which AuNP probe was used as a second indicator of a closed tube SYBR safe-LAMP assay. This simplified and inexpensive technique allowed rapid interpretation within a few minutes with high sensitivity and specificity. This additional step of AuNP-probe could improve and simplify the closed tube LAMP assay to empower asymptomatic leishmaniasis surveillance in HIV patients. Indeed, the probe based on biotinylated loop primer could be used as simplified and generalized nanoprobes in any LAMP assays for specific verification, whereas SYBR safe could be used for general screening. Therefore, this one-step LAMP assay is practical and useful for field diagnostics to point-of-care settings that is easily delivered to end-users, particularly in low to middle income countries.

## Results

### Specific AuNP-LAMP probe assay

**Figure 1:**
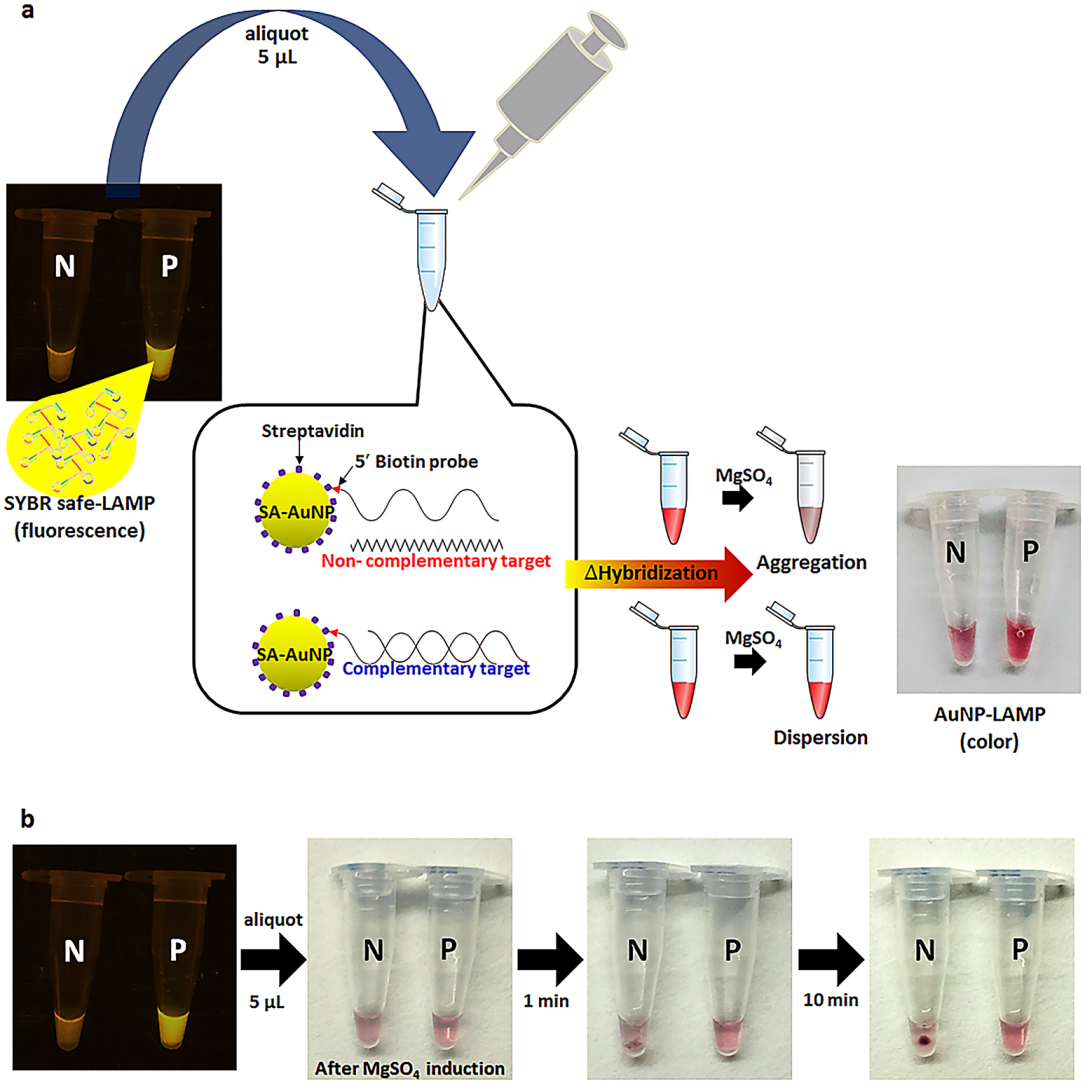
Procedure of LAMP detection using gold nanoparticles (AuNPs) probe in one-step SYBR safe and AuNP-LAMP assay, (**a**) schematic representation of experimental principle and signal detection to visualize LAMP products by post hybridization of probe-streptavidin conjugated AuNPs (probe-SA-AuNPs), (**b**) colorimetric and precipitate interpretation of probe-SA-AuNPs in postamplification LAMP mixture after MgSO_4_ induction.

A schematic of the hybridization principle of LAMP probe-streptavidin conjugated AuNPs (SA-AuNP) and colorimetric precipitate induction is shown in Fig. 1a. The 5 µL of LAMP reaction of closed tube SYBR safe LAMP method is transferred to a new tube for colorimetric precipitate detection by post hybridization of AuNP-probe. This process includes hybridization of the complementary single strand probe-SA-AuNP to complementary LAMP target, induction of colorimetric and precipitate signal by MgSO_4_ and colorimetric precipitate detection by the naked eye. The hybridization of the single strand probe-SA-AuNP forms the polymeric network of cross-linked AuNPs that does not trigger the aggregation by salt induction, leading to unchanged red color of the reaction mixture. Conversely, an absence of the LAMP targets leads to the aggregation of free probe-SA-AuNPs and the reaction mixture turns pale purple with dark precipitates. The aggregation began after the reaction had been induced with MgSO_4_ salt. One minute later, the sediment of precipitates was formed then dense aggregation was observed at 10 min. SYBR safe, as the first indicator, in LAMP reaction showed no interference on colorimetric precipitate induction of AuNP probes (Fig. 1b).

### Optimization of LAMP probe conjugated AuNPs

**Figure 2:**
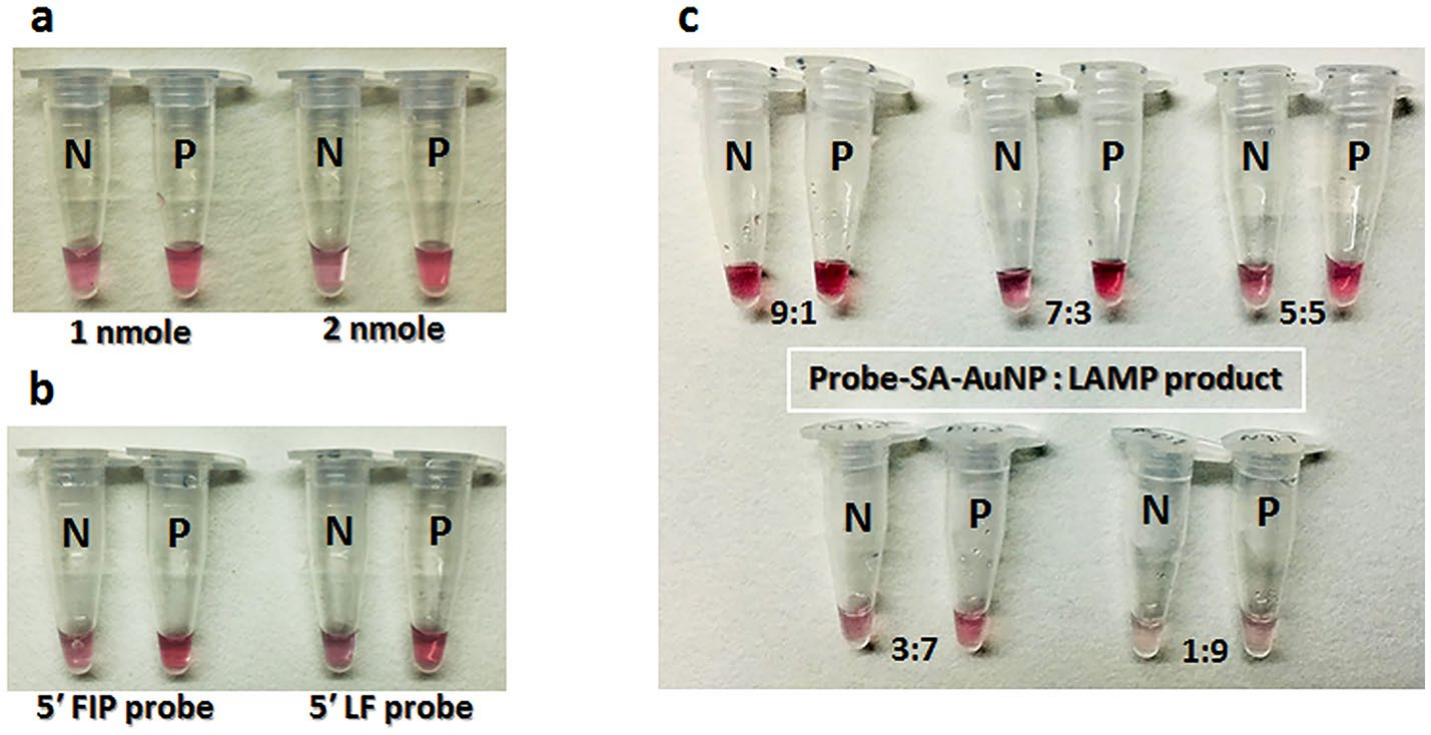
Optimization of probe-SA-AuNPs to detect *Leishmania* DNA, (**a**) effect of probe-SA-AuNPs concentration, (**b**) effect of probe-SA-AuNPs type, (**c**) effect of ratios between probe-SA-AuNP and LAMP product. Abbreviations of variable names: no template control (N), positive template control (P), forward inner primer (FIP), loop forward primer (LF).

**Figure 3:**
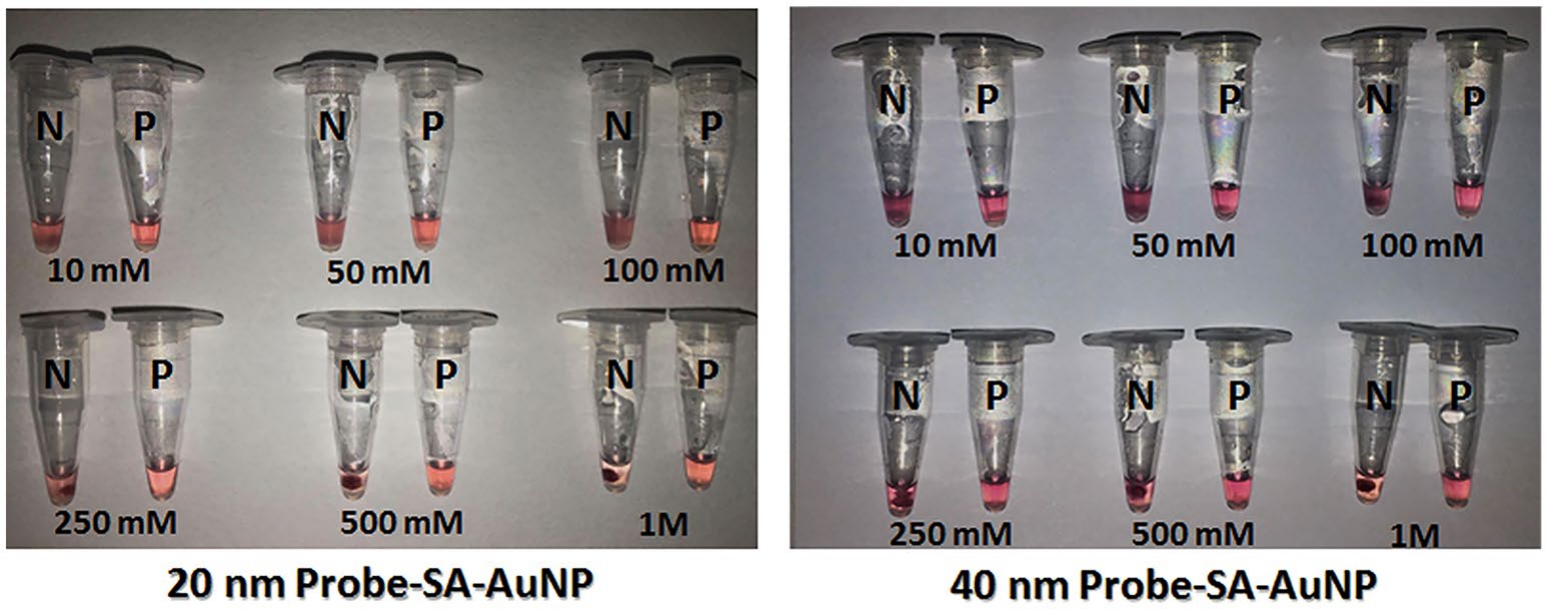
Effect of MgSO_4_ concentration (10 to 1000 mM at a fixed volume) on inducing aggregation of 20 and 40 nm probe-SA-AuNPs. Abbreviations of variable names: no template control (N), positive template control (P).

No difference in color was observed when one and two nmole probes were hybridized to the LAMP amplicons (Fig. 2a). Furthermore, unambiguous color was visualized between different types of the AuNP probes, FIP and LF; that specifically located in loop region but varied in size and length (Fig. 2b). One-to-one volume ratio (1:1) of LAMP product and AuNP probe presented the clearest interpretation that a positive result remained deep red and a negative result was induced to pale purple with precipitate (Fig. 2c). An aggregation of probe-SA-AuNP could be induced at all concentrations of MgSO_4_ salt in both 20 and 40 nm sizes (Fig. 3). However, clear cut color was interpreted in the mixture of 40 nm probe-SA-AuNPs. The precipitates were observed at 10 to 100 mM; whereas, the precipitates were sedimented when the salt concentration was increased to 250 mM. According to clearer interpretation of color and precipitate, 40 nm SA-AuNP was chosen as a standard size to link with the biotin-LAMP probe.

**Figure 4:**
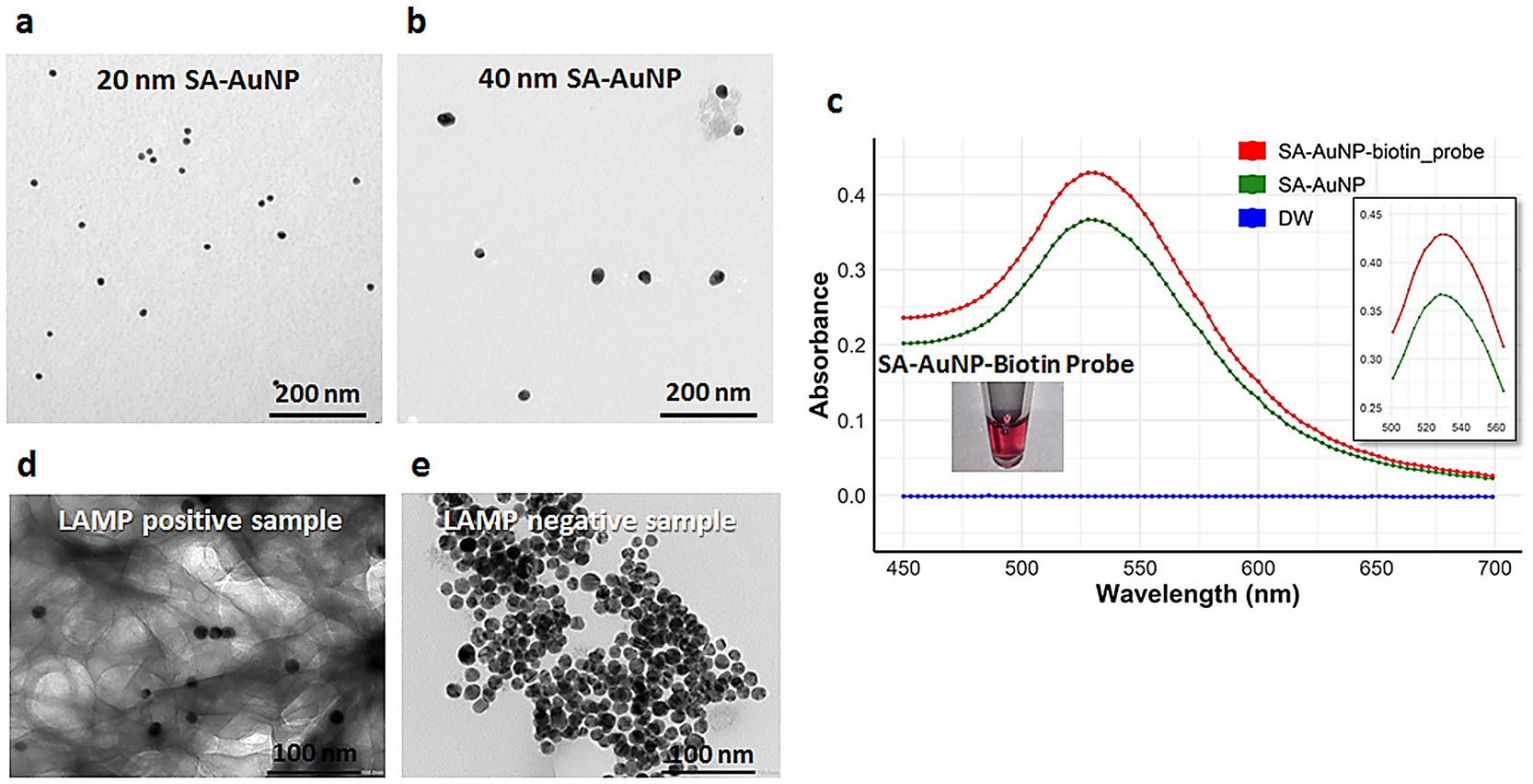
Characterization of SA-AuNPs and probe-SA-AuNPs, (**a**) transmission electron microscopy (TEM) imaging of SA-AuNPs at 20 nm size, (**b**) TEM imaging of SA-AuNPs at 40 nm size, (**c**) absorbance spectra of colloidal SA-AuNPs and probe-SA-AuNPs (SA-AuNP-biotin probe), (**d**) TEM of positive samples with probe-SA-AuNPs, (**e**) TEM of negative samples with probe-SA-AuNPs.

The synthesized SA-AuNP (20 and 40 nm) and LAMP products hybridized with probe-SA-AuNPs were visualized using TEM (Fig. 4). The 20 and 40 nm SA-AuNPs showed a round configuration differing by size (Fig. 4a and b). The optical properties demonstrated major peak of absorbance of unlinked streptavidin-AuNP (40 nm) at 527 nm. Upon linking the biotin-LAMP probe on the SA-AuNP, the major peak shifted to 530 nm as shown in Fig. 4c. Regarding salt induction using MgSO_4_, the dispersion of probe-SA-AuNPs was observed in the solution with complementary LAMP targets (Fig. 4d); whereas, an aggregation of free probe-SA-AuNP was found in the reaction without LAMP products (Fig. 4e).

### LOD and specificity

**Figure 5:**
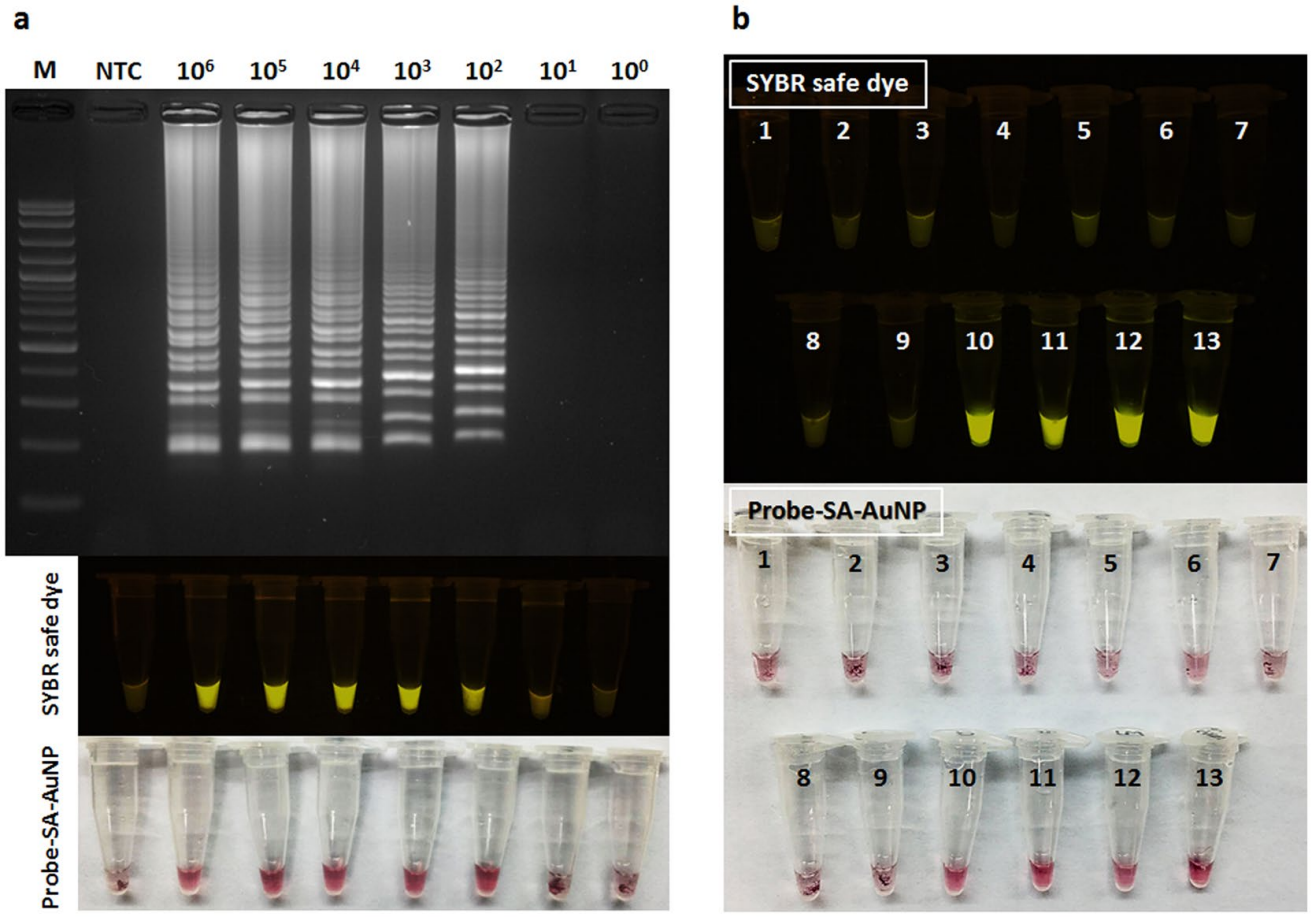
Sensitivity and specificity of one-step SYBR safe and AuNP-LAMP assay, (**a**) LOD of the assay under gel electrophoresis, fluorescent and color visualization (ten fold serial dilution; 10^6^–10^0^ parasites/mL): 100 bp ladder (M), no template control (NTC), (**b**) LAMP detection of different pathogens’ DNA based on SYBR safe dye and probe-SA-AuNP: tube 1; nuclease-free water, tube 2; human genomic DNA, tubes 3–13; *E. coli*, *G. intestinalis*, *T. vaginalis*, *P. falciparum*, *N. gonorrhea*, *S. pyogenes*, *S. flexneri*, *T. evansi*, *L. donovani*, *L. martiniquensis* and *L. siamensis*, respectively.

A variety of multiple bands of stem-loop DNA structures were exhibited by gel electrophoresis indicating successful amplification of LAMP products. Concordance of LOD was observed between visual inspection of fluorescent SYBR and colorimetric precipitate AuNP probe, and gel electrophoresis, in that 10^2^ parasites/mL (0.0147 ng/µL) could be detected (Fig. 5a). No cross amplifications were detected except *T. evansi* when non*Leishmania* pathogens’ DNA were tested against the LAMP primers using the one-step SYBR safe and AuNP-LAMP assay (Fig. 5b).

### Evaluation of sensitivity and specificity of SYBR safe and AuNP-LAMP assay

Eighty-five extracted DNA samples from buffy coat including leishmaniasis cases (*n* = 17) and uninfected cases (*n* = 68) were used to evaluate and validate the one-step SYBR safe and AuNP-LAMP assay. Compared with the reference standard of nested PCR, the sensitivity and specificity of AuNP-LAMP probe were 94.1 (*n* = 16; 95% CI, 71.3 to 99.9%) and 97.1% (*n* = 66; 95% CI, 89.8 to 99.6%), respectively (Table 1). Additionally, the sensitivity of two closed tube LAMP assays (fluorescence and color) were the same as colorimetric precipitate detection using post hybridization of AuNP-LAMP probes, 94.1% (*n*_*fluorescent*_ = 16, *n*_*colorimetric*_ = 16; 95% CI, 71.3 to 99.9%, respectively), nonetheless, the specificity was higher in only the colorimetric closed tube method, 98.5% (*n* = 67; 95% CI, 92.1 to 100.0%). Thus, the AuNP-LAMP probe showed significant results that were in almost perfect agreement when compared with fluorescent and colorimetric closed tube LAMPs (*P*-value < 0.001, < 0.001; Kappa = 1.00, 0.89, respectively).

**Table 1:** Sensitivity and specificity of postamplification detection using specific AuNP-LAMP probes and prereaction detection using other nonspecific dye indicators in LAMP assays to detect *Leishmania* DNA among patients with asymptomatic leishmaniasis.

LAMP methods	No. (*n*) of samples with nested PCR^a^		(%) Sensitivity (95% CI)	(%) Specificity (95% CI)	(%) PPV (95% CI)	(%) NPV (95% CI)
	Positive	Negative				
**Postamplification detection**						
*AuNP-LAMP probes*			94.1 (71.3–99.9)	97.1 (89.8–99.6)	88.9 (65.3–98.6)	98.5 (92.0–100.0)
Positive	16	2				
Negative	1	66				
**Prereaction detection**						
*Fluorescent closed tube*^b^			94.1 (71.3–99.9)	97.1 (89.8–99.6)	88.9 (65.3–98.6)	98.5 (92.0–100.0)
Positive	16	2				
Negative	1	66				
**Prereaction detection**						
*Colorimetric closed tube*^c^			94.1 (71.3–99.9)	98.5 (92.1–100.0)	94.1 (71.3–99.9)	98.5 (92.1–100.0)
Positive	16	1				
Negative	1	67				

^a^Total number of positive results was based on nested PCR (reference standard method); ^b^SYBR safe initially mixed in the reaction before salt induction of postamplification detection was used as a fluorescent indicator; ^c^SYBR green I was used as a color indicator.

## Discussion

The highly sensitive and selective determination of *Leishmania* infection in asymptomatic HIV patients was achieved using dual indicators (fluorescent SYBR safe and colorimetric precipitate gold-nanoparticle probe) in one-step LAMP method. The LAMP assay could successfully detect *Leishmania* DNA from blood by emission of fluorescent SYBR safe, in prereaction mixture, and colorimetric precipitate of LAMP specific AuNP-probe, in postamplification mixture. The polymeric network of cross-linked AuNPs was formed, leading to unchanged red color after salt induction, when AuNP-probe hybridized to the target of LAMP amplicons^33,34^. In contrast, the reaction interpreted pale purple color with precipitates in the absence of LAMP targets^25,35,36^. Regarding the absence of polymeric network, the probe-SA-AuNPs were induced to aggregation and sedimented in negative samples and no template control (NTC) resulting in color change from red to pale purple^38^. The SYBR safe dye in closed tube LAMP assay did not interfere in the hybridizing and aggregating of AuNPs-probe, even though, the dye was remained in the postamplification mixture of the AuNP-LAMP method.

The successful functionalized SA-AuNPs to the specific LAMP probe was demonstrated by the shift of absorption peak that was usually observed upon functionalizing bare AuNPs and SA-AuNPs with biomolecule ligands such as streptavidin or thiol, as well as oligonucleotide probes^27–29,39,40^. In spite of the unambiguous evaluation using 20 nm and 40 nm SA-AuNPs, 40 nm nanoparticles interpreted clearer color and precipitate result. Owing to the larger size of the 40 nm probe-SA-AuNP structure, higher mass weight of aggregated AuNPs-probe could be physically formed after salt induction. False interpretations of color change and precipitates increased under higher concentration of MgSO_4_^26,28,40^. Therefore, appropriate MgSO_4_ concentration and mixture ratio of probe-SA-AuNP should be concerned to limit false aggregation and gain the best visualization of color and precipitate. Finally, simple and rapid step of postamplification preparation of closed tube SYBR safe LAMP assay, 5 min at 65 °C, is required to clearly interpret specific result of LAMP detection by AuNPs-probes hybridization.

The sensitivities of SYBR safe and AuNP-probe detection were limited to 10^2^ parasites/mL or 0.0147 ng/µL, exhibiting ten times more sensitivity than those of the MG and SYBR safe assays^11,13^, and was equal to a simplified closed tube assay, SYBR green I^23^. Similar to related studies of Sriworarat et al.^11^ and Sukphattanaudomchoke et al.^23^, the pan-*leishmania* primers could amplify and cross react with DNA from *Trypanosoma* sp., a closely related blood protozoan. Otherwise, trypanosomiasis exhibited different clinical presentations; therefore, the cross reaction would not significantly lead to misdiagnosis^11^. To overcome the limitation of cross-reactivity, additional LAMP primers specific to *Trypanosoma* spp. could be used to amplify the co-existence of these two blood protozoa in co-infected endemic areas^41^. Multiplex gene detection in the LAMP assay maybe possible when the fluorescent dye labeling LAMP primers are specifically synthesized^10^ or when a specific colorimetric LAMP microfluidic chip has been developed^42,43^. The sensitivity (*n* = 16, 94.1%) and specificity (*n* = 66, 97.1%) of colorimetric precipitate using post hybridization of AuNP-probe were almost equal to those of the closed tube LAMP assays using fluorescent SYBR safe dye (94.1 and 97.1%) and colorimetric SYBR green I (94.1 and 98.5%) when nested PCR was used as the reference standard for detecting *Leishmania* DNA in patients with asymptomatic HIV. Furthermore, the kappa statistical test indicated almost perfect agreement between these LAMP detecting methods. Although only buffy coat samples were used in this study, samples such as saliva or urine should be further validated to confirm feasibility of these one-step SYBR safe and AuNP-LAMP assay.

Compared with rapid diagnostic test kits (RDT) such as malaria and VL strip tests, the LAMP reaction requires initial DNA for the detection. Therefore, DNA extraction is inevitable in all nucleic acid amplification test (NAAT) techniques. Although RDT is simpler and faster than NAAT, the sensitivity and specificity of RDT are lower. Thus, the most sensitive NAATs are commonly used to detect causative agents. Adversely, NAATs may not be affordable to use at local health service centers due to the inconvenient requirement of costly sophisticated instruments and well-trained lab technicians. Contrastingly, LAMP requires basic equipment, whereas local trained health care workers could perform the assay according to its simple operating procedure. To overcome the limitations of LAMP in the field sites, simplified methods have been successfully modified and adapted to the assays such as selective colorimetric and fluorescent dyes^11,13,23,44^, lyophilized form^45^, paper-based devices^46^ and dye capsules^15,18^. In the meantime, ongoing development of the new approaches encourages the use of LAMP for point-of-care (POC) in low resource settings such as health services as well as field sites; not only for screening but also to confirm diagnostic level. This work demonstrated the application of LAMP for *Leishmania* detection among asymptomatic HIV patients commonly harboring low parasite levels. A double detection step using a specific LAMP probe and DNA-binding fluorescent dye can be performed simultaneously, taking approximately 75 to 80 min after adding DNA samples. The LAMP procedure is faster than the standard method, nested PCR, which takes about 8 to 10 h working in the laboratory^3^. Additionally, the assay requires only a simple heating block or noninstrument nucleic acid amplification (NINA) that is comparatively much cheaper than PCR; and particularly, qPCR^47^. By replacing with the simplified boiling extraction method^11^, the price per reaction could be reduced about 70%. To achieve the shortened process of the reaction time, new loop primers (LF, loop-forward and LB, loop-backward) could be further introduced in the prereaction preparation^48^. This study supported that LAMP assays could be adapted with several modifications and applications. For example, the DNA-binding SYBR safe indicator could be compatible with a specific colorimetric precipitate AuNP-probe that can be used as a second clear-cut indicator for LAMP amplicon control. In addition, LAMP and other detective devices such as microfluidic chips^42,43^, multiplex gene detection^10^ and lateral flow dipstick (LFD)^41,49^ could be applied in the future. Thus, the LAMP assay is a very promising method that helps early diagnosis and treatment to reduce severe and prevent progression of the disease.

## Conclusion

In this study, we have developed one-step LAMP assay using dual indicators to detect *Leishmania* in buffy coat of asymptomatic HIV patients by fluorescence and colorimetric precipitate. The additional step of AuNPs-probes hybridization in postamplification preparation of closed tube SYBR safe LAMP assay required 5 min at 65 °C to clearly interpret the specific results and could detect *Leishmania* DNA as low as 10^2^ parasites/mL (0.0147 ng/µL). The sensitivities and specificities of fluorescent SYBR safe dye and AuNP-probe indicators were equal, which were as high as 94.1 and 97.1%, respectively. The one-step SYBR safe and AuNP-LAMP assay are suitable for multipurpose *Leishmania* detection, for example, direct evaluation or screening in field environments using closed tube SYBR safe method to reduce contamination prone^13,19^ or, re-evaluation and verification using post hybridization of AuNPs-probe method to control and assure the first detection^26,40^. In addition, the remaining LAMP products could be further quantified by a distance-based paper device^46^. These applications could improve and additionally simplify the closed tube SYBR safe dye LAMP assay to empower asymptomatic leishmaniasis surveillance in HIV patients. Furthermore, the probe based on biotinylated loop primer could be used as simplified and generalized nanoprobes in any LAMP assays for specific verification, whereas SYBR safe could be used for general screening. Hence, the assay was not limited to only *Leishmania* diagnosis and could be used as an alternative method in addition to traditional PCR detection, especially in areas with limited resources. The one-step SYBR safe and AuNP-LAMP assay is reliably fast and practical for field diagnostics to point-of-care settings that is easily delivered to end-users, especially in low to middle income countries.

## Materials and methods

### Ethics statement

Participants aged more than 18 years were enrolled in the study. Written informed consent was received from all participants before sample collection and anonymous analysis. All methods were carried out in accordance with relevant guidelines and regulations. This study was approved by the Ethics Committee of the Royal Thai Army Medical Department (IRBRTA 952/2562).

### DNA preparation for clinical samples and *Leishmania* parasites culture

EDTA anticoagulated blood samples (8 mL) were collected from eligible participants visiting the HIV Clinic at Satun Hospital, Satun Province every six months for follow-up testing and to receive antiretroviral therapy (ART). To separate the plasma and buffy coat, the whole blood sample was centrifuged at 900 × g for 10 min and then kept at –20 °C until further DNA extraction.

A total culture of 10^7^ promastigotes of *L. siamensis* (MON-324; MHOM/TH/2010/TR), *L. martiniquensis* (MON-229; WHOM/TH/2011/PG) and *L. donovani* (MHOM/ET/67/HU3) using Schneider’s *Drosophila* medium (Sigma, St. Louis, MO, USA) supplemented with 20% heat inactivated fetal bovine serum (GE Healthcare, Chicago, IL, USA) at 26 °C were harvested and washed three times with phosphate buffered saline (PBS) before DNA extraction.

The buffy coat was extracted for DNA using the Geneaid™ DNA Isolation Kit (blood) (New Taipei, Taiwan), whereas genomic DNA of each species of *Leishmania* parasites was extracted using the DNeasy Extraction Kit (tissue) (Qiagen, Hilden, Germany) according to manufacturer protocols. The concentration and quality of the extracted DNA were analyzed by Nanodrop spectrophotometer (Denovix, Wilmington, DE, USA) at 260/280 and 260/230 ratios and kept at –20 °C until further use.

### PCR amplification for *Leishmania* DNA detection

Nested PCR described by Manomat et al.^3^ was used to amplify the internal transcribed spacer (ITS1) region of the ribosomal RNA (rRNA) gene of *Leishmania* using a FlexCycler2 Thermocycler (Analytik Jena, Jena, Germany). Promastigotes’ DNA of *L. martiniquensis* (WHOM/TH/2011/PG) was used as a positive control. Positive PCR amplicons were purified and sent to Bionics Co. Ltd. (Seoul, South Korea) for sequencing. The sequencing chromatograms were validated and multiple-aligned with reference *Leishmania* strains retrieved from GenBank using BioEdit, Version 7.0.1.

### Biotin loop-mediated isothermal amplification (LAMP) probe design

The loop forward (LF) region of 18s rRNA LAMP amplicons, described by Sriworarat et al.^11^, was chosen to develop specific probes. Specific probes of the LF region, located between F2 and F1C regions, was manually designed, complementary to the loop region, using consensus sequence to avoid any nonspecific region presented. A consensus sequence was made from a highly conserved 18s ribosomal RNA (rRNA) gene of nine different *Leishmania* species including GenBank accession numbers: KJ467218.1, KF041809.1, KF041806.1, KF041804.1, KF041801.1, KF041797.1, KF302752.1, KF302746.1 and KF041798.1. A LAMP probe with less negative value for Gibb’s free energy (∆G) in dimers and hairpin loop formations was chosen to ensure optimality. In addition, the thermodynamic properties were calculated using OligoCalc Software (http://biotools.nubic.northwestern.edu/OligoCalc); whereas, the specificity of the probe was verified against human and any other organisms’ DNA using Basic Local Alignment Search Tool (BLAST) analysis (https://blast.ncbi.nlm.nih.gov/Blast.cgi). Finally, the LF probe was modified by labeling with biotin at 5′ region and custom synthesized by Bionics Co. Ltd. (Seoul, South Korea). The biotin labeling LAMP probes including newly designed LF and FIP are shown in Table 2.

**Table 2:** Biotin-LAMP probe and LAMP primer sequences (Sriworarat et al.^11^) used in this study.

Type name	Sequence (5′ − 3′)	Length (bp)
**Probes**		
LF	biotin– TGT GGT GCC ATT CCG TCA	18
FIP	biotin– GTC AAA TTA AAC CGC ACG CTC CAC GGG GGA GTA CGT TCG CAA	42
**Primers**		
FIP	GTC AAA TTA AAC CGC ACG CTC CAC GGG GGA GTA CGT TCG CAA	42
BIP	TCA ACA CGG GGA ACT TTA CCA GAT CAC CAC CAT TCA GGG AAT CGA	45
F3	CGA AAG CTT TGA GGT TAC AGT CT	23
B3	CAA ACA AAT CAC TCC ACC GAC	21

### Loop-mediated isothermal amplification assay

*LAMP amplification* A total volume of 25 µL LAMP reaction was prepared as described by Sriworarat et al.^11^. The LAMP primers are shown in Table 2. Briefly, each LAMP reaction mixture consisted of 40 pmol of FIP and BIP primers, 10 pmol of F3 and B3 primers, 1× Thermopol buffer, 8 mM MgSO_4_, 1.4 mM of each dNTP (Biotechrabbit, Hennigsdorf, Germany), 0.8 M betaine (Sigma-Aldrich), 8 U of *Bst* DNA Polymerase Large Fragment (New England Biolabs, Ipswich, MA, USA) and 2 µL of DNA template. In addition, 1× SYBR™ Safe DNA Gel Stain (Thermo Fisher Scientific, Waltham, MA, USA) was added to each reaction mixture during prereaction preparation^13^ as the nonspecific fluorescent dye indicator of LAMP products. The reaction was incubated at 65 °C for 75 min and then heated at 80 °C for 10 min to inactivate the reaction.

To prepare a closed tube LAMP using SYBR green I (Thermo Fisher Scientific), the reaction was prepared in a total volume of 25 µL as described by Sukphattanaudomchoke et al.^23^, that, before amplification, 1 µL of 1000× SYBR green I was inwardly dotted at the tube lid and a piece of parafilm® M (Bermis, Oshkosh, WI) was placed covering two thirds of the tube opening to form a semiclosed layer between the dye and LAMP reaction mixture. The remaining one third open space of parafilm layer allowed the SYBR green I to pass through and stain the LAMP amplicons after inactivating the amplification. The dye from the lid of the reaction tube was briefly spun down using a minicentrifuge (Hercuvan, Cambridge, UK) to mix with the reaction mixture after incubating.

*LAMP visualization* After incubating, the LAMP amplicons of target DNA were analyzed and confirmed based on direct visual inspection using either a blue light (BL) or an ultraviolet light UV transilluminator. For fluorescent interpretation, a positive amplification showed vivid fluorescent emission, whereas no fluorescent emission was observed without amplification. Regarding validation of LAMP amplification, 5 µL of reaction mixture was electrophoresed on 2% agarose gel stained with SYBR™ Safe (Thermo Fisher Scientific) and visualized using an UV transilluminator to determine a mixture of various lengths of the stem-loop DNA of LAMP products.

### Synthesis and optimization of LAMP probe conjugated AuNPs

A particle diameter of 20 and 40 nm of colloidal AuNPs stabilized in phosphate buffer that had been conjugated with streptavidin; OD = 10, were commercially synthesized by Kestrel Bioscience Thailand Co. Ltd., Thailand. Streptavidin-AuNPs were previously diluted at 1:1 with phosphate buffer and subsequently mixed with 100 µM 5′-biotin labeling LF and FIP probes at 1:10 ratio by vigorous vortex in a dark at room temperature for 15–30 s to obtain 1 nmole streptavidin-AuNP-5′-biotin-probes structure (OD = 0.5). The LAMP probe conjugated AuNPs were then kept in the dark at 4 °C until used.

The ratio of the optimum hybridization condition was modified from Suebsing et al.^28^. The hybridization to detect *Leishmania* LAMP amplicons was conducted in a total volume of 15 µL at 65 °C for 5 min. Briefly, the LAMP probe conjugated AuNPs and LAMP reaction mixture were mixed at different ratios, i.e., 9:1, 7:3, 5:5, 3:7 and 1:9. As the salt concentration could induce aggregation, various concentrations of MgSO_4_ were further tested at 10 mM to 1 M in a 5 µL fix volume of postamplification mixture (15 µL) to determine the best concentration to visually detect color and colloidal change. After incubating, a positive result that free AuNP-LAMP probe-amplicon complexes were formed in the reaction remained deep red, whereas pale purple with dark insoluble precipitate was observed in the absence of LAMP products.

Streptavidin-AuNP (20 and 40 nm) and reaction mixture of the positive and negative samples were transferred onto a carbon-coated copper grid and dried at ambient temperature to measure the sizes and the structure effects of AuNP-LAMP probes using a JEM-2100 transmission electron microscope (TEM), respectively (JEOL Ltd., Tokyo, Japan). The 40 nm streptavidin-AuNP (SA-AuNP) was subsequently used as standard size to link to the hybridized LAMP probe before being further optimized in the study. Additionally, wavelengths of the maximum absorption (λ_max_) including SA-AuNPs and probe-SA-AuNPs were measured by UV–visible spectrum analysis using an EnSight™ multimode plate reader (PerkinElmer®, Waltham, MA, USA).

### Detection limits (LOD) and specificity

Twenty microliters of *Leishmania* parasites at the mid log phase were collected and resuspended with 20 µL of phosphate buffered saline (PBS) containing 0.2% glutaraldehyde (GE, Healthcare, USA). The total parasite densities (parasites/mL) were counted using a Neubauer Chamber at 400× magnification under light microscope. To determine the sensitivity of LAMP primers, purified genomic DNA of *L. siamensis* were serially diluted tenfold from 10^6^ to 10^0^ parasites/mL that were equivalent to 1.147 µg/µL to 1.147 pg/µL, respectively, using a Nanodrop spectrophotometer. The experiments were performed in triplicate, and nuclease-free water was used as negative control.

The LAMP reactions were tested against human genomic DNA extracted from buffy coat and non*Leishmania* DNA including genomic DNA extracted from *Escherichia coli*, *Shigella flexneri*, *Streptococcus pyogenes*, *Neisseria gonorrhea*, *Plasmodium falciparum*, *Trichomonas vaginalis, Trypanosoma evansi* and *Giardia intestinalis* to ensure that AuNP-LAMP probes were specific to *Leishmania* and cross amplification with other pathogens was unlikely. Extracted DNA of three *Leishmania* species including *L. siamensis*, *L. martiniquensis* and *L. donovani* were used as positive controls and, nuclease-free water was used for no template control (NTC).

### Evaluation of sensitivity and specificity of SYBR safe and AuNP-LAMP assay

Sensitivity and specificity of AuNP-LAMP probes were determined using a total of 85 genomic DNA from clinical samples, consisting of 18 confirmed asymptomatic VL and 67 uninfected cases. Diagnosis of VL was confirmed when nested PCR targeting ITS1 region was positive and DNA sequence of the PCR amplicons was identical to rRNA gene of *Leishmania*. Results from nested PCR are considered a reference standard method due to the unavailability of any gold standard^50^. These data were used to validate and evaluate the sensitivity and specificity of SYBR safe-LAMP and AuNP-LAMP based on these dual indicators used for LAMP assay. The strength of the agreement was determined between prereaction detection, including simplified colorimetric fluorescent closed tube (SYBR green I), fluorescent closed tube (SYBR safe), and postamplification detection using specific AuNP-LAMP probes. The kappa statistical test at 95% confidence intervals (CI) and *P*-value < 0.05 were used to assess different LAMP assays. A *P*-value < 0.05 was considered statistically significant. The analysis was performed using STATA, Version SE14 (Stata Corporation, College Station, TX, USA).

## Supplementary Information

Supplementary Information.

## Data Availability

The datasets generated during the current study are available from the corresponding author on reasonable request.
