# Supplementary material for: Development of loop-mediated isothermal amplification (LAMP) assay using SYBR safe and gold-nanoparticle probe for detection of Leishmania in HIV patients
Source: Sci Rep. 2021 Jun 9;11:12152. doi: 10.1038/s41598-021-91540-5 (PMC8190085; doi:10.1038/s41598-021-91540-5)

Supplementary Information

Development of loop-mediated isothermal amplification (LAMP) assay using SYBR safe and gold-nanoparticle probe for detection of *Leishmania* in HIV patients

Toon Ruang-areerate[1*], Charanyarut Sukphattanaudomchoke[2], Thanyapit Thita[1], Saovanee Leelayoova[1], Phunlerd Piyaraj[1], Mathirut Mungthin[1], Patcharapan Suwannin[3], Duangporn Polpanich[4], Tienrat Tangchaikeeree[3], Kulachart Jangpatarapongsa[3], Kiattawee Choowongkomon[5] and Suradej Siripattanapipong[2*]

[1]Department of Parasitology, Phramongkutklao College of Medicine, Bangkok, 10400, Thailand. [2]Department of Microbiology, Faculty of Science, Mahidol University, Bangkok, 10400, Thailand. [3]Center for Research and Innovation, Faculty of Medical Technology, Mahidol University, Bangkok 10700, Thailand. [4]National Nanotechnology Center (NANOTEC), National Science and Technology Development Agency (NSTDA), Pathum Thani 12120, Thailand. [5]Department of Biochemistry, Faculty of Science, Kasetsart University, Bangkok, 10900, Thailand

*Address correspondence to Toon Ruang-areerate, Department of Parasitology, Phramongkutklao College of Medicine, Bangkok, 10400, Thailand, and Suradej Siripattanapipong, Department of Microbiology, Faculty of Science, Mahidol University, Bangkok, 10400, Thailand. E-mails: youangtr@yahoo.com and suradej.sir@mahidol.ac.th

Figure S1. Original images of the gels shown in Figure 5.

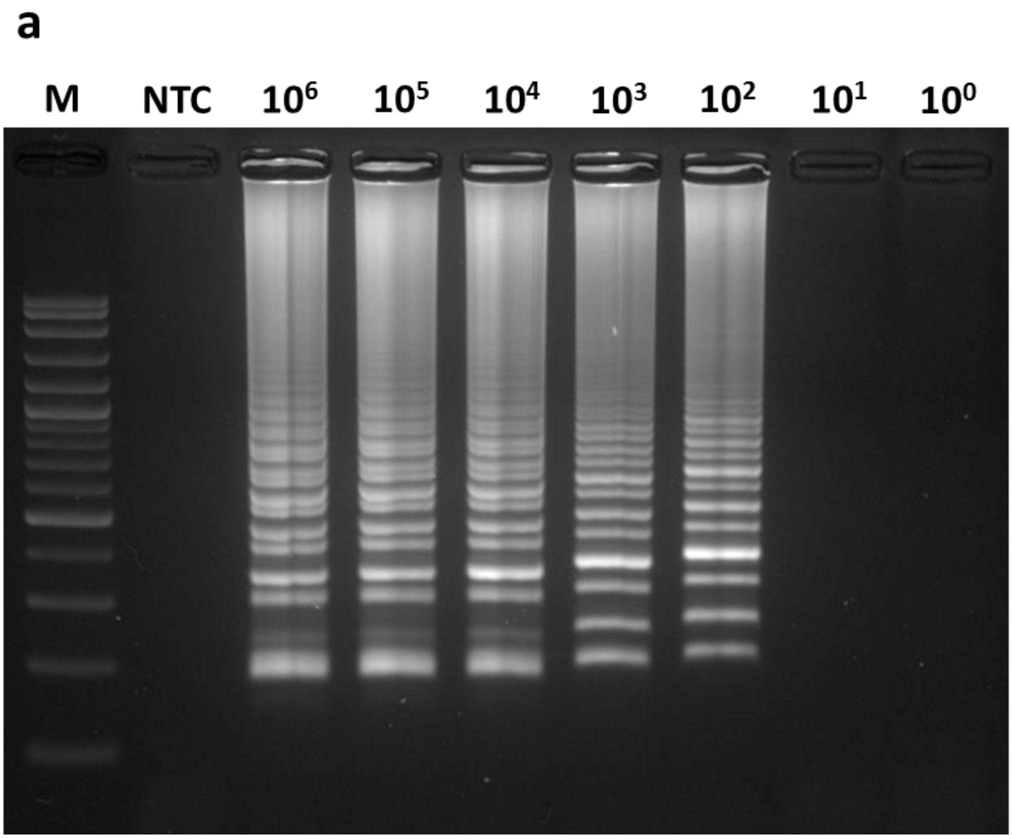

Supplement: Supplementary file 1 — Supplementary Information. [file 41598_2021_91540_MOESM1_ESM.pdf]
